# Characterization of DNA lesions associated with cell-free DNA by targeted deep sequencing

**DOI:** 10.1186/s12920-021-01040-8

**Published:** 2021-07-28

**Authors:** Seung-Ho Shin, Woong-Yang Park, Donghyun Park

**Affiliations:** 1 GENINUS Inc, Seoul, 05836 Korea; 2 Samsung Genome Institute, Samsung Medical Center, Seoul, 06351 Korea; 3 Department of Molecular Cell Biology, Sungkyunkwan University School of Medicine, Suwon, 16419 Korea

**Keywords:** Circulating cell-free DNA, Targeted deep sequencing, DNA damages, Cytosine deamination errors

## Abstract

**Background:**

Recently, a next-generation sequencing (NGS)-based method has been used for the successful detection of circulating tumor DNA (ctDNA) in various cancer types. Thus, the use of NGS on liquid biopsies will improve cancer diagnosis and prognosis. However, the low-allelic fraction of ctDNA poses a challenge for the sensitive and specific detection of tumor variants in cell-free DNA (cfDNA). To distinguish true variants from false positives, the characteristics of errors that occur during sample preparation and sequencing need to be elucidated.

**Methods:**

We generated capture-based targeted deep sequencing data from plasma cfDNA and peripheral blood leucocyte (PBL) gDNA to profile background errors. To reveal cfDNA-associated DNA lesions, background error profiles from two sample types were compared in each nucleotide substitution class.

**Results:**

In this study, we determined the prevalence of single nucleotide substitutions in cfDNA sequencing data to identify DNA damage preferentially associated with cfDNA. On comparing sequencing errors between cfDNA and cellular genomic DNA (gDNA), we observed that the total substitution error rates in cfDNA were significantly higher than those in gDNA. When the substitution errors were divided into 12 substitution error classes, C:G>T:A substitution errors constituted the largest difference between cfDNA and gDNA samples. When the substitution error rates were estimated based on the location of DNA-fragment substitutions, the differences in error rates of most substitution classes between cfDNA and gDNA samples were observed only at the ends of the DNA fragments. In contrast, C:G>T:A substitution errors in the cfDNA samples were not particularly associated with DNA-fragment ends. All observations were verified in an independent dataset.

**Conclusions:**

Our data suggested that cytosine deamination increased in cfDNA compared to that in cellular gDNA. Such an observation might be due to the attenuation of DNA damage repair before the release of cfDNA and/or the accumulation of cytosine deamination after it. These findings can contribute to a better understanding of cfDNA-associated DNA damage, which will enable the accurate analysis of somatic variants present in cfDNA at an extremely low frequency.

**Supplementary Information:**

The online version contains supplementary material available at 10.1186/s12920-021-01040-8.

## Background

The presence of circulating tumor DNA (ctDNA) in blood samples from patients with cancer has been identified due to the detection of somatic tumor variants in cell-free DNA (cfDNA) [1–4]. Furthermore, cfDNA analysis has drawn enormous attention for its clinical potential as a non-invasive method to detect and profile tumor mutations [5]. In addition to the non-invasiveness of a simple blood draw, since blood samples represent the profile of many anatomical locations, cfDNA analysis is expected to overcome the limitations of tissue biopsies involving the overrepresentation of a subpopulation enriched at a sampling site [2, 6]. cfDNA analysis has been made possible by targeted deep sequencing methods based on next-generation sequencing (NGS) technologies [7].

However, several challenges remain with respect to the clinical application of cancer diagnosis and monitoring through cfDNA analysis by targeted deep sequencing [5, 8]. Of these, the most critical technical challenge is posed by the low allelic frequency of tumor-derived cfDNA fragments, which is often far below 1% [9, 10]. To detect such low-frequency variants, the detection method has to be not only extremely sensitive but also extremely specific. Achieving high sensitivity requires a high depth of unique coverage [11], which can be achieved cost-effectively by target enrichment methods based on PCR amplification or hybridization-based capture. A depth of unique coverage of approximately 500 × is usually reported to be sufficient to profile genetic alterations in tumor specimens [12], but many cfDNA sequencing studies have aimed for a higher depth of unique coverage of 2000–10,000 × [13, 14].

In addition to a high depth of unique coverage, cfDNA sequencing requires a lower level of systemic background substitution errors [15], as it is difficult to identify a true variant present at a frequency below or at the same level as that of errors. As the number of false variants is correlated with the number of tests, a high specificity is required for panels designed to examine large genomic regions [15]. For this reason, conventional panel sequencing has limitations with respect to detecting tumor variants present at an allele frequency of less than 1‒5%, which is sufficient to detect somatic alterations in tumor specimens but not in cfDNA. To overcome these problems, studies have reported several diverse strategies, including methods for reducing background errors by exploiting various statistical models for data analysis and/or the data generation scheme; for example, by including a unique molecular identifier (UMI) [16, 17]. Newman et al. (2016) demonstrated a ~ 0.004% detection rate for tumor variants utilizing a UMI combined with several statistical models for suppressing substitution errors [10]. Phallen, Jillian et al. demonstrated that a UMI-based assay can detect ~ 0.1% tumor variants with more than 99.99% specificity [9]. Technical replicates of sequencing libraries have been used to remove prevailing background errors and to detect low-level somatic mutations [18]. Physical isolation of DNA clones from the NGS substrate after the sequencing procedure was also attempted to distinguish true variants from sequencing errors [19].

In addition to these methods for filtering out errors from sequencing data, many studies have sought to suppress the occurrence of errors or characterize the causes of these errors. Many of these errors were caused by DNA damage and were introduced during DNA extraction, library construction, or sequencing procedures [15, 20]. Of the various forms of DNA damage, deamination and oxidation-induced DNA damage are especially well documented [21–23]. It is important to characterize the etiology of background errors in sequencing data to make the attenuation of errors by avoiding their cause feasible. For instance, errors due to oxidative DNA lesions could be reduced using anti-reactive agents or by modifying experimental conditions to minimize DNA oxidation [23]. We also reported that the oxidation of guanine residues may cause C:G>A:T errors during DNA fragmentation, which can be suppressed by lowering the acoustic shearing power [15]. Another prominent DNA damage is cytosine deamination, which converts cytosine to uracil. Given that uracil hydrogen bonds with adenine in a subsequent replication step, a C:G>T:A transition during PCR amplification results in C:G>T:A errors in sequencing data. In addition to these studies, sequencing platform-, hybrid selection-, storage-, and chemical-induced errors were also reported [10, 15, 24, 25].

Although many studies have identified errors artificially introduced during experimental procedures, sequencing errors caused by cfDNA-associated DNA damage have not yet been systematically examined. We hypothesized that cfDNA has more DNA lesions than cellular genomic DNA for two reasons. First, cellular genomic DNA is constantly repaired to remove DNA lesions; this might not be properly executed after the initiation of cell death. Given that cfDNA release is known to be associated with cell death, cfDNA might carry accumulating DNA damage prior to or during cell death. Second, despite the relatively short half-life of cfDNA, DNA damage might occur during its circulation after release, and thus, not be properly repaired.

In this study, we sought to identify cfDNA-associated sequencing errors. From duplicated targeted deep sequencing data sets generated from five healthy donors, we estimated and compared the substitution error rates between paired cfDNA and gDNA samples across 12 substitution classes. The results from the analysis were validated in another data set from our previous study involving the detection of circulating tumor DNA in patients with lymphoma [12]. Like the characterization of sequencing errors due to other artificial DNA damage, the identification of cfDNA-associated errors will be helpful for improving the detection specificity of ctDNA in cfDNA sequencing.

## Methods

### Sample collection and DNA extraction

This study and its protocol were approved by the Research Ethics Committee of the Samsung Medical Center and ST. Mary’s Hospital. Whole blood samples were collected in two Cell-Free DNA™ BCT tubes (Streck Inc., Omaha, NE, USA) from five healthy volunteers and EDTA tubes from eleven prostate cancer patients. To estimate the degree of technical variability between repeated experiments, we made two duplicated pairs of plasma and peripheral blood leucocyte (PBL) samples in healthy volunteer cohort. While plasma was obtained by performing three centrifugation steps using increasing centrifugal forces, PBLs were isolated from the initial centrifugation step. After the preparation steps, plasma and PBL samples were stored at − 80 °C until DNA extraction. Genomic DNA (gDNA) was isolated from blood samples using a QIAamp DNA mini kit (Qiagen, Santa Clarita, CA, USA). Plasma DNA was obtained from 3.5 to 4.5 mL of plasma using a QIAamp Circulating Nucleic Acid Kit (Qiagen, Santa Clarita, CA, USA). The concentration, purity, and fragment size of the DNA were measured using previously reported methods [14].

### Generation of sequencing data

As part of this study, we generated targeted sequencing data for paired cfDNA and gDNA samples from five healthy donors. In addition, the sequencing data from our previous study [12] on patients with lymphoma were analyzed to validate our observations, which had been also generated for paired cfDNA and gDNA samples from the same patients.

Purified gDNA was sonicated into 400–500 bp fragments using Covaris S2 (Covaris Inc. Woburn, MA, USA). The PBLs and plasma DNA libraries were prepared using a KAPA Hyper Prep Kit (Kapa Biosystems, Woburn, MA, USA), as described previously [14]. Hybrid selection for target enrichment was performed using customized panels. Target enrichment baits (Agilent, Santa Clara, CA, USA) targeting 82 cancer-related genes were used in healthy cohort, while 60 prostate cancer-related gene panel (Twist Biosciences, San Francisco, CA, USA) was customized for the prostate cancer cohort.

As previously described [14], libraries were diluted to a concentration of 2 nM, based on DNA concentration and average fragment size, and then pooled in equal volumes. The pooled libraries were denatured and then subjected to cluster amplification according to the manufacturer’s instructions (Illumina, San Diego, CA, USA). Flow cells were sequenced in the 100 bp paired-end mode using HiSeq 2500 v3 Sequencing-by-Synthesis Kits (Illumina, San Diego, CA, USA) and then analyzed using RTA v.1.12.4.2 or later.

### Sequencing data processing

All generated reads from each sample were aligned to the hg19 human reference with BWA-mem (v0.7.17) [26] to create BAM files. Samtools (v1.9) [27] was used to sort and index BAM files. The MarkDuplicate module in the Picard (v2.19.0) package was used to categorize reads into UMI families, and then a home-built python (v2.7.10) script was used for digital error suppression (DES). The DES method was based on previously reported studies [10] with a minor modification. Briefly, a degenerated four-base barcode adjacent to sample barcode for multiplexing was sequenced as part of the index read and used for UMI. Based on UMI combined with the chromosomal positions of DNA fragment, sequence reads were categorized into UMI families to distinguish true somatic mutations from PCR and/or sequencing errors. The base quality score recalibration (BQSR) process was performed using the GATK package (v4.1.0.0) [28]. All sequencing statistics were collected from Samtools and the Picard QC processes (Additional file 1: Table S1). The typical MarkDeduplicate method was used to compare with the DES method.

### Analysis of background error profiling

We identified background errors in the sequencing data by removing putative variants and bases with a quality score. The following selection steps were used: (1) the reference allele was excluded in the analysis; (2) low quality bases were filtered out (Phread quality score < 30); (3) genomic locations with less than 500 × sequencing coverage depth was removed; (4) if the variant was present at an allele frequency above 1% in either cfDNA or PBL sample, it was excluded from the analysis. For the analysis of sequencing data from patients with lymphoma samples, we also removed somatic tumor variants from the plasma DNA samples. The detailed process for identifying somatic tumor variants has been described in our previous study [12]. After the removal of somatic and germline variants, we identified 12 classes of substitution errors in each sample and conducted statistical comparative analysis using the R program [29]. Mutational signature analysis performed by signal web analysis tool (http://signal.mutational signatures.com) and SBS mutational signature was selected for analyzing the signatures contributions from COSMIC database [30].

### Background error analysis based on the position on the DNA fragment

Background errors across all substitution classes were assigned at each position relative to the DNA breakpoint (1‒50 bp). The errors were then grouped in the 5 bp bin to compare the relative error rates. To extract the location and substitution class of errors from sequence reads, we used an in-house python (v2.7.10) script and a pysam (v0.15.2) library.

## Results

### Increased C:G>T:A substitution errors in plasma cfDNA

We assumed that the increase in DNA damage in cfDNA compared to that in cellular genomic DNA (gDNA) would result in increased errors in cfDNA sequencing data compared to gDNA data. DNA lesions altering base pairing during DNA replication are known to be one of the primary causes of sequencing errors in a variety of contexts [31, 32]. To characterize DNA lesions preferentially present in cfDNA, we generated sequencing data for cfDNA and cellular gDNA using the exact same method (see Methods). The only exception was that cellular gDNA was fragmented using mild acoustic shearing, such that DNA damage was minimized, as we previously reported. Fragmentation-related DNA lesions in cellular gDNA samples, if present, might prevent cfDNA-associated damages from being detected. Thus, although we could not demonstrate the absence of a particular cfDNA-associated damage due to this limitation, a significant elevation in the error rate for cfDNA samples should reflect cfDNA-associated DNA lesions.

**Fig. 1:**
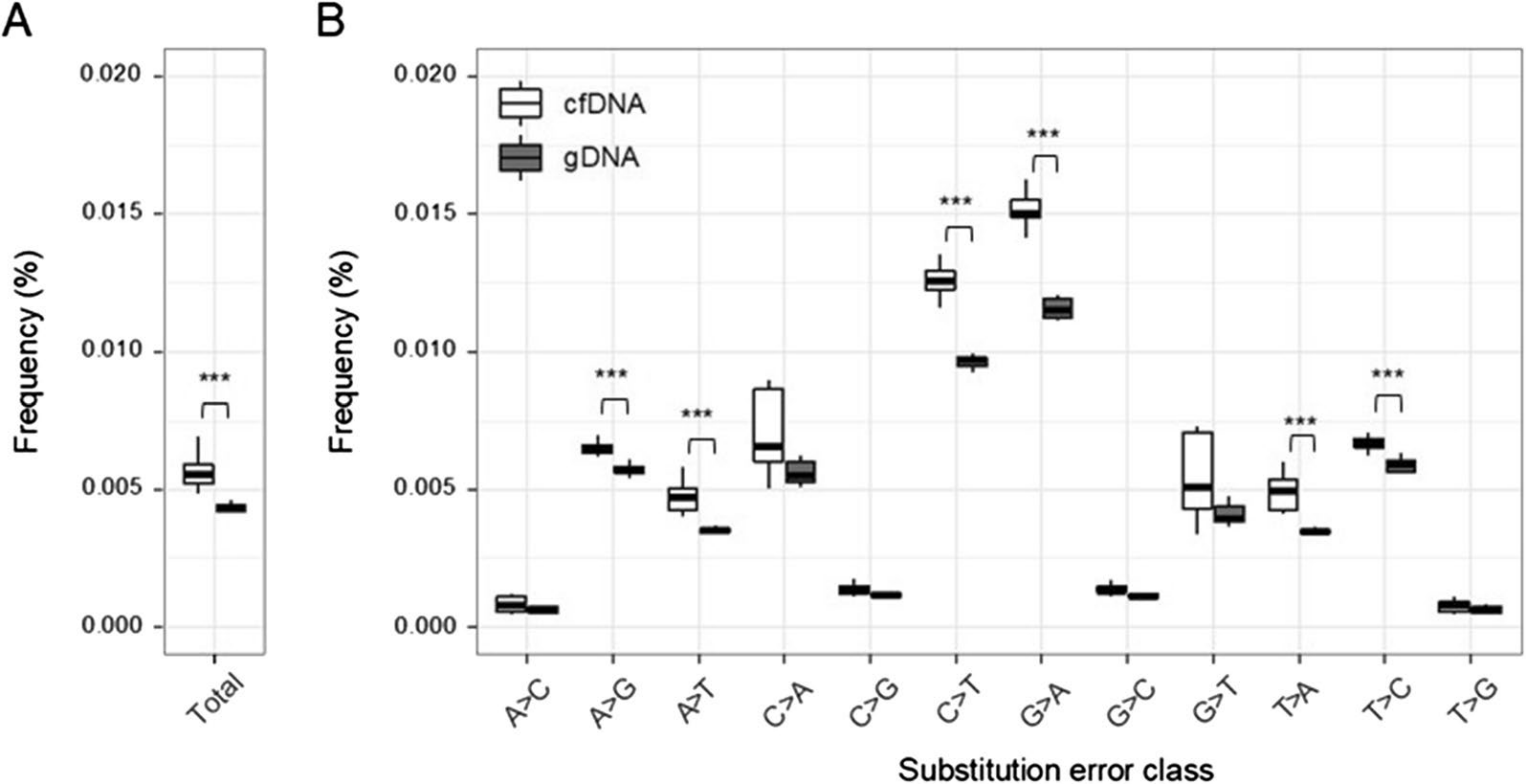
Comparison of the error rates in sequencing data of cell-free DNA (cfDNA) and cellular genomic (gDNA) samples. The box plots display the distribution of the mean error rates **A** in total and **B** for the twelve substitution classes for the cfDNA and cellular gDNA samples

First, after identifying errors in cfDNA and gDNA sequencing data, we compared the total substitution error rates of cfDNA and gDNA. Our results showed significantly more errors in the cfDNA sequencing data than those in the gDNA sequencing data. The average substitution error rates of cfDNA and gDNA were 0.00574 $\pm$ 0.0003% and 0.00271% $\pm$ 0.0002%, respectively (*p*-value = 2 × 10^–4^; Fig. 1A). Next, we measured the error rates across all 12 substitution classes. Although the overall patterns across the 12 substitution classes were similar for the cfDNA and gDNA data, we observed the differences in the cfDNA and gDNA error rates in particular substitution classes (Fig. 1B). We found that C>T and G>A substitution errors were significantly more frequent in cfDNA than in gDNA (Bonferroni adjusted *p*-value < 0.01; Fig. 1B). To rule out the possibility that technical variability might contribute this difference, we replicated the data set for each sample. The differences in average error rates between the two data sets were less than 0.0003% across all substitution classes, clearly demonstrating that technical variability hardly explains the significant elevation of C:G > T:A errors in cfDNA (Additional file 2: Figure S1). Furthermore, the increase in C:G > T:A substitution errors in cfDNA was also observed in an independent data set (Bonferroni adjusted *p*-value < 0.02; Additional file 3: Figure S2) generated from 20 patients with lymphoma. To negate the possibility of artifacts due to differential DNA damage between cfDNA and cellular gDNA caused by the fixative in the blood collection tube, we performed the same analysis using samples collected in EDTA tubes. Consistently, the C:G > T:A error was elevated in cfDNA compared to that in gDNA (*p*-value < 0.01; Additional file 4: Figure S3). Our data strongly suggest that the increase in C:G > T:A errors in the cfDNA samples was not due to technical artifacts but rather due to sample type-associated attributes.

These results demonstrated that C:G > T:A substitution errors increased in cfDNA compared to cellular gDNA, primarily leading to an overall increase in the error rate for cfDNA. Our results indicated that cfDNA was relatively prone to a cytosine lesion (i.e., likely cytosine deamination) that base-paired with adenine instead of guanine.

### Random genomic position of cfDNA-associated C:G>T:A errors

Sequencing errors are largely dependent on their context; thus, error rates vary dramatically across different chromosomal positions [13]. The context dependency of errors is relevant not only for sequencing platform-associated errors but also for certain types of errors due to DNA lesions [15, 33]. For instance, the frequency of C:G > A:T transversions due to guanine oxidation dramatically changes depending on their context, which also varies with the etiology of guanine oxidation [15, 23]. Thus, we speculated if the increased C:G>T:A errors in cfDNA were associated with a subset of chromosomal positions, indicating a context dependency.

**Fig. 2:**
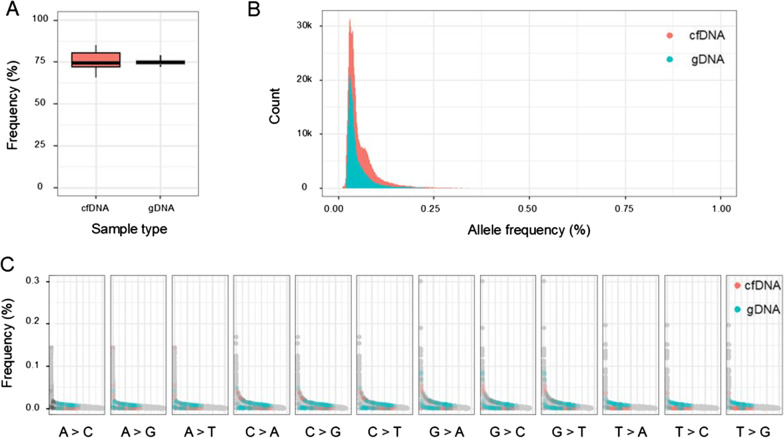
Position-specific error rates in sequencing data. **A** The percentage of error-free positions in each sample was calculated and presented as a box-plot for each group. **B** The counts of the position-specific error rates for each group are plotted on the y-axis. At each position, the average background error rate is calculated for each group. **C** The position-specific error rates across the 12 substitution classes is displayed in descending order. They are gray dots, except when they differ significantly between groups, in which case they are red for cfDNA and green for gDNA

First, we compared the fraction of error-free positions between cfDNA and gDNA. The fraction of error-free positions is 75 $\pm$ 0.72% for plasma cfDNA and 76 $\pm$ 1.96% for gDNA (Fig. 2A). Second, we calculated the error rate at every genomic position of target regions and compared the distribution of errors in cfDNA and gDNA. Substitution errors were measured at 199,538 out of 202,429 bp, excluding positions where any sample did not achieve 500 × depth of coverage. Consistent with the elevated average error rate in cfDNA, the distribution of cfDNA position-specific error rates shifted to the right compared to that in gDNA (Fig. 2B). Then, we performed a *t*-test to identify positions where the error rates of the two groups were significantly different. Based on the reference sequence, the test was performed for 52,174 positions for A>N, 48,239 positions for C>N, 48,062 positions for G>N, and 51,063 positions for T>N substitution errors. We only found 843 positions where position-specific error rates differed between the groups, which is 0.42% of the total positions tested (Fig. 2C). Among the 843 positions tested, 20.2% (171/843) were C:G>T:A substitutions. Although the error rate of C:G>T:A substitutions was the highest among all the substitution classes, the number of positions displaying a significant difference in error rate between plasma and genomic DNA was not greater than that in other substitution classes. The cfDNA error rate was higher than that for gDNA at 75 out of 171 positions, which were highly likely to be false positives.

To examine if these handful of positions contributed significantly to the elevated cfDNA error rate, we again profiled error rates across 12 substitution classes after excluding these 843 positions. Removal of these positions did not change the error rates across 12 substitution classes. The total error rate decreased by less than 4% for both cfDNA and gDNA, which means that position specific errors have little effect on the elevated cfDNA error rate. Moreover, the C:G>T:A error rate in cfDNA samples was not lower than the error rate before the exclusion (1.38 × 10^–5^).

Taken together, our results indicated that the increase in C:G>T:A errors in cfDNA was not predominantly due to a small fraction of the positions. Instead cfDNA-associated cytosine lesions might randomly arise without a strong sequence dependency.

### Independence of cfDNA-associated C:G>T:A errors from DNA breakpoints

**Fig. 3:**
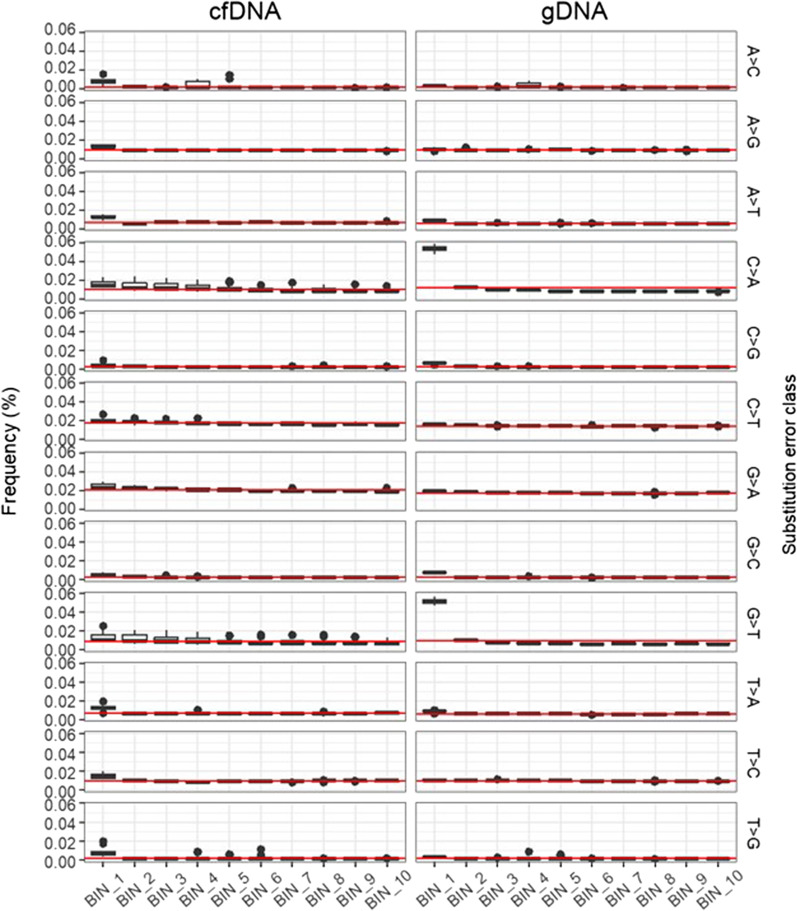
Background error rates according to the positions relative to the DNA breakpoint. The error rates were binned by every 5 bp based on their nucleotide position in the reads and plotted across the 12 substitution classes for each group. The red line indicates the average error rates of each substitution class

As DNA lesions might be relatively frequent near DNA breakpoints, as shown in previous reports, we investigated whether the substitution error rates depended on the location relative to the DNA breakpoints. To evaluate how substitution errors differ depending on their position in DNA fragments, the substitution error rate at every 5-bp interval from 1 to 50 bp (positions relative to the DNA breakpoint) of the sequencing reads were assigned to bins 1–10, respectively. In both samples types, we observed that the average substitution error rate was higher in the first bin than other bins (*p*-value = 1.90 × 10^–11^ and 1.98 × 10^–8^ for cfDNA and gDNA, respectively). On the contrary, we did not find a significant difference in the C:G > T:A substitution errors between the first and second bins of cfDNA (Fig. 3). Although C:G > T:A substitution errors increased slightly near the ends of the DNA fragments compared to their middle regions (Fig. 3), this pattern was not significantly different between the groups; indicating that cfDNA-associated cytosine lesions were distributed more or less evenly across the DNA fragment. These patterns were also observed in the other data set (Additional file 5: Figure S4).

## Discussion

In this study, we demonstrated that there were more C:G>T:A errors in sequencing data generated for cfDNA compared to that generated for cellular gDNA due to cfDNA-associated lesions, most likely due to cytosine deamination. A number of studies have reported that cytosine deamination can causes C:G>T:A substitutions [20, 22]. While it is a frequently occurring DNA lesion caused by endogenous cellular processes, C:G>T:A errors are far more frequently caused by technical artifacts occurring during experimental procedures, including sample preparation and storage. Since DNA fragmentation is essential for constructing sequencing libraries from cellular gDNA, identification of cfDNA-associated errors through comparison with the errors in cellular gDNA is hampered by its greater level of artificial sequencing errors due to DNA fragmentation. Previously, we reported a DNA fragmentation protocol that minimizes DNA lesions by lowering the ultrasonic acoustic energy [15]. By leveraging this protocol, we could identify cfDNA-associated DNA lesions, which would not be evident had we used a standard protocol (Additional file 6: Figure S5).

In fact, the average error rate of cellular gDNA samples prepared using the standard protocol was higher than that of the cfDNA sample, this was reversed when the optimized protocol was used. As shown in Fig. 1, the error rates across all substitution classes were never higher in cfDNA than in gDNA. When we calculated the error rates by location in the DNA fragments, the gDNA error rate for any of the substitution classes was not significantly higher near DNA breakpoints, suggesting that DNA damage associated with DNA fragmentation was minimal. One exception was the C:G>A:T error rate, which was significantly higher in the first 5 bp of the gDNA fragments (Bonferroni adjusted *p*-value < 0.01; Fig. 3, Additional file 5: Figure S4). These data indicated the presence of guanine oxidation due to acoustic shearing near DNA breakpoints, even though we used a mild shearing condition. Thus, we excluded the ends of the DNA fragments and compared the C:G>A:T error rate between cfDNA and cellular gDNA. Although it did not differ significantly between the groups, we could not conclude whether the absence of cfDNA-associated C:G>A:T errors was due to limitations of our analysis. In addition to C:G>T:A, cfDNA-associated errors might occur in other substitution classes. For instance, we found that A:T>T:A errors were slightly, yet significantly, elevated in cfDNA samples. However, except for C:G>T:A substitution errors, the difference in substitution errors was not significant in our analysis of an independent data set (Fig. 1, Additional file 3: Figure S2). In addition, when we performed mutational signature analysis using single base substitution (SBS) mutation signature from the COSMIC database [30], we observed that only SBS6, one of several defective DNA mismatch repair signatures, was significantly elevated in cfDNA error profiles (9.53% vs. 7.70%; Bonferroni adjusted *p*-value < 0.01; Additional file 7: Table S2). On the other hand, no difference was observed in other defective DNA mismatch repair signatures. Although these results were consistent with our primary observation, it still remained to identify the mechanism underlying the error rate elevated in cfDNA.

Although our results coherently indicated the elevation of C>T error rate in cfDNA samples, there are also limitations in our study. As we used a hybrid selection-based targeted sequencing method with the Illumina sequencer, our study needs to be validated further using other methods. The technical advancements in library construction or sequencing platforms that decrease the background error level in the sequencing data may result in the uncovering of subtle differences that are masked by background noise in this study. In addition, it would be better to test the findings using various pre-analytic procedures. Owing to the possibility of low and varied DNA damage between cfDNA and cellular gDNA caused by the unknown chemical in Streck tubes, we generated an additional dataset from samples collected using EDTA tubes. Although we found a consistent pattern of the C:G>T:A error in samples collected in non-fixative tubes, it is still difficult to directly compare datasets from two different collection tubes owing to variations attributed to other factors in the dataset, such as blood donors and capture methodology (see Methods **for details on library preparation). Further studies on DNA damage involved in these pre-analytical procedures can provide us a better understanding of DNA lesions associated with plasma cfDNA. Nonetheless, the results from the additional dataset obtained using EDTA tubes indicated that the fixative reagent did not affect our findings.

In this study, we used UMIs to reduce PCR errors. UMIs have been suggested to suppress background errors and increase the depth of unique coverage. However, the effect of the UMIs in our study was marginal; the mean frequency for all substitution errors was decreased by only 32% in gDNA (0.0064% to 0.0044%) and 22% in cfDNA (0.0073% to 0.0057%). These results were because the average number of progenies in each unique template in our sequencing data was only 2.79, which was not enough to take full advantage of UMIs for suppressing PCR errors. In the in silico error suppression method using UMIs, the number of progenies of PCR duplicates is a major factor affecting the performance of the error suppression method. To increase the effect of UMI, strategies for increasing PCR progenies, such as improvement of capture uniformity, reduction of PCR bias, and increase in data size, need to be considered.

## Conclusions

By analyzing the substitution errors in sequencing data, we found that C:G>T:A errors were higher in cfDNA than in gDNA. These cfDNA-associated errors were not related to specific locations in the genome. We also found that C:G>T:A substitution errors were still significantly increased after removing the position on the DNA fragment with a significant difference in error rate between cfDNA and gDNA. The frequency of C:G>T:A substitution errors did not change with location relative to the DNA breakpoints. In conclusion, we identified cfDNA-associated substitution errors that were likely to be caused by cytosine deamination damage. Although there may be fewer cfDNA-associated DNA lesions than those created during experimental procedures, the characterization of cfDNA-associated errors is critical for detecting somatic mutations that are present at a low frequency, especially considering recent rapid improvements that reduce technical artifacts.

## Supplementary Information

**Additional file 1: Table S1.** Sequencing data statistics.

**Additional file 2: Figure S1.** Comparison of the error rates between duplicates. The mean error rates across the 12 substitution classes between duplicate experiments are shown as box plots.

**Additional file 3: Figure S2.** Comparison of error rates in the sequencing data of cfDNA and cellular gDNA samples. The box plots display the distribution of mean error rates (left panel) in the 12 substitution classes and (right panel) in total from the cfDNA and cellular gDNA samples. While the data in Figure 1 were obtained from healthy volunteers, the data shown in this figure were obtained from lymphoma patients.

**Additional file 4: Figure S3.** Comparison of 12 substitution error rates between plasma cfDNA and cellular gDNA samples collected in non-fixative EDTA tube. The box plots display the distribution of mean error rates in each substitution error class. ^***^; Asterisks indicates statistically significant difference (p-value ≤ 0.01).

**Additional file 5: Figure S4.** Background error rates near the DNA break point. The error rates for the first three 5 bp-bins near the DNA break points from the cfDNA and gDNA samples were calculated and compared. Data generated independently from (a) healthy volunteers and (b) lymphoma patients show similar patterns across all substitution classes.

**Additional file 6: Figure S5.** Differences in error rates due to DNA fragmentation. The mean error rates across the 12 substitution classes are shown as box plots for the mild shearing condition (left panel) and the standard condition (right panel).

**Additional file 7: Table S2.** Contribution of mutational signatures.

## Data Availability

The sequencing data analyzed during the current study are deposited in European Nucleotide Archive (ENA) under accession number “ERP129660”. Reference genome (hg19) used in this study can be obtained from the UCSC databases (https://hgdownload.soe.ucsc.edu/).

## Abbreviations

BQSR: Base quality score recalibration
cfDNA: Cell-free DNA
ctDNA: Circulating tumor DNA
DES: Digital error suppression
gDNA: Genomic DNA
NGS: Next generation sequencing
PBL: Peripheral blood leucocyte
UMI: Unique molecular identifier

## References

[1] Bettegowda C, Sausen M, Leary RJ, Kinde I, Wang Y, Agrawal N, Bartlett BR, Wang H, Luber B, Alani RM (2014). Detection of circulating tumor DNA in early- and late-stage human malignancies. Sci Transl Med.

[2] Abbosh C, Birkbak NJ, Wilson GA, Jamal-Hanjani M, Constantin T, Salari R, Le Quesne J, Moore DA, Veeriah S, Rosenthal R (2017). Phylogenetic ctDNA analysis depicts early-stage lung cancer evolution. Nature.

[3] Forshew T, Murtaza M, Parkinson C, Gale D, Tsui DW, Kaper F, Dawson SJ, Piskorz AM, Jimenez-Linan M, Bentley D (2012). Noninvasive identification and monitoring of cancer mutations by targeted deep sequencing of plasma DNA. Sci Transl Med.

[4] Diehl F, Li M, Dressman D, He Y, Shen D, Szabo S, Diaz LA, Goodman SN, David KA, Juhl H (2005). Detection and quantification of mutations in the plasma of patients with colorectal tumors. Proc Natl Acad Sci USA.

[5] Siravegna G, Mussolin B, Venesio T, Marsoni S, Seoane J, Dive C, Papadopoulos N, Kopetz S, Corcoran RB, Siu LL (2019). How liquid biopsies can change clinical practice in oncology. Ann Oncol.

[6] Wan JCM, Massie C, Garcia-Corbacho J, Mouliere F, Brenton JD, Caldas C, Pacey S, Baird R, Rosenfeld N (2017). Liquid biopsies come of age: towards implementation of circulating tumour DNA. Nat Rev Cancer.

[7] Chen M, Zhao H (2019). Next-generation sequencing in liquid biopsy: cancer screening and early detection. Hum Genom.

[8] Cohen JD, Li L, Wang Y, Thoburn C, Afsari B, Danilova L, Douville C, Javed AA, Wong F, Mattox A (2018). Detection and localization of surgically resectable cancers with a multi-analyte blood test. Science.

[9] Phallen J, Sausen M, Adleff V, Leal A, Hruban C, White J, Anagnostou V, Fiksel J, Cristiano S, Papp E (2017). Direct detection of early-stage cancers using circulating tumor DNA. Sci Transl Med.

[10] Newman AM, Lovejoy AF, Klass DM, Kurtz DM, Chabon JJ, Scherer F, Stehr H, Liu CL, Bratman SV, Say C (2016). Integrated digital error suppression for improved detection of circulating tumor DNA. Nat Biotechnol.

[11] Chung J, Son DS, Jeon HJ, Kim KM, Park G, Ryu GH, Park WY, Park D (2016). The minimal amount of starting DNA for Agilent's hybrid capture-based targeted massively parallel sequencing. Sci Rep.

[12] Shin SH, Kim YJ, Lee D, Cho D, Ko YH, Cho J, Park WY, Park D, Kim SJ, Kim WS (2019). Analysis of circulating tumor DNA by targeted ultra-deep sequencing across various non-Hodgkin lymphoma subtypes. Leuk Lymphoma.

[13] Newman AM, Bratman SV, To J, Wynne JF, Eclov NC, Modlin LA, Liu CL, Neal JW, Wakelee HA, Merritt RE (2014). An ultrasensitive method for quantitating circulating tumor DNA with broad patient coverage. Nat Med.

[14] Park G, Park JK, Son DS, Shin SH, Kim YJ, Jeon HJ, Lee J, Park WY, Lee KH, Park D (2018). Utility of targeted deep sequencing for detecting circulating tumor DNA in pancreatic cancer patients. Sci Rep.

[15] Park G, Park JK, Shin SH, Jeon HJ, Kim NKD, Kim YJ, Shin HT, Lee E, Lee KH, Son DS (2017). Characterization of background noise in capture-based targeted sequencing data. Genome Biol.

[16] Schmitt MW, Kennedy SR, Salk JJ, Fox EJ, Hiatt JB, Loeb LA (2012). Detection of ultra-rare mutations by next-generation sequencing. Proc Natl Acad Sci USA.

[17] Kinde I, Wu J, Papadopoulos N, Kinzler KW, Vogelstein B (2011). Detection and quantification of rare mutations with massively parallel sequencing. Proc Natl Acad Sci USA.

[18] Kim J, Kim D, Lim JS, Maeng JH, Son H, Kang H-C, Nam H, Lee JH, Kim S (2019). The use of technical replication for detection of low-level somatic mutations in next-generation sequencing. Nat Commun.

[19] Yeom H, Lee Y, Ryu T, Noh J, Lee AC, Lee H-B, Kang E, Song SW, Kwon S (2019). Barcode-free next-generation sequencing error validation for ultra-rare variant detection. Nat Commun.

[20] Chen L, Liu P, Evans TC, Ettwiller LM (2017). DNA damage is a pervasive cause of sequencing errors, directly confounding variant identification. Science.

[21] Chen G, Mosier S, Gocke CD, Lin MT, Eshleman JR (2014). Cytosine deamination is a major cause of baseline noise in next-generation sequencing. Mol Diagn Ther.

[22] Do H, Wong SQ, Li J, Dobrovic A (2013). Reducing sequence artifacts in amplicon-based massively parallel sequencing of formalin-fixed paraffin-embedded DNA by enzymatic depletion of uracil-containing templates. Clin Chem.

[23] Costello M, Pugh TJ, Fennell TJ, Stewart C, Lichtenstein L, Meldrim JC, Fostel JL, Friedrich DC, Perrin D, Dionne D (2013). Discovery and characterization of artifactual mutations in deep coverage targeted capture sequencing data due to oxidative DNA damage during sample preparation. Nucleic Acids Res.

[24] Laehnemann D, Borkhardt A, McHardy AC (2016). Denoising DNA deep sequencing data-high-throughput sequencing errors and their correction. Brief Bioinform.

[25] Wong SQ, Li J, Salemi R, Sheppard KE, Do H, Tothill RW, McArthur GA, Dobrovic A (2013). Targeted-capture massively-parallel sequencing enables robust detection of clinically informative mutations from formalin-fixed tumours. Sci Rep.

[26] Li H, Durbin R (2010). Fast and accurate long-read alignment with Burrows-Wheeler transform. Bioinformatics.

[27] Li H, Handsaker B, Wysoker A, Fennell T, Ruan J, Homer N, Marth G, Abecasis G, Durbin R (2009). The Sequence Alignment/Map format and SAMtools. Bioinformatics.

[28] McKenna A, Hanna M, Banks E, Sivachenko A, Cibulskis K, Kernytsky A, Garimella K, Altshuler D, Gabriel S, Dalyl M (2010). The Genome Analysis Toolkit: a MapReduce framework for analyzing next-generation DNA sequencing data. Gen Res.

[29] Team RC: R: A language and environment for statistical computing. 2013.

[30] Tate JG, Bamford S, Jubb HC, Sondka Z, Beare DM, Bindal N, Boutselakis H, Cole CG, Creatore C, Dawson E (2019). COSMIC: the catalogue of somatic mutations in cancer. Nucleic Acid Res.

[31] McBride TJ, Preston BD, Loeb LA (1991). Mutagenic spectrum resulting from DNA damage by oxygen radicals. Biochemistry.

[32] Valentine MR, Rodriguez H, Termini J (1998). Mutagenesis by peroxy radical is dominated by transversions at deoxyguanosine: evidence for the lack of involvement of 8-oxo-dG1 and/or abasic site formation. Biochemistry.

[33] Schirmer M, Ijaz UZ, D'Amore R, Hall N, Sloan WT, Quince C (2015). Insight into biases and sequencing errors for amplicon sequencing with the Illumina MiSeq platform. Nucleic Acids Res.

