# Supplementary material for: Characterization of DNA lesions associated with cell-free DNA by targeted deep sequencing
Source: BMC Med Genomics. 2021 Jul 28;14:192. doi: 10.1186/s12920-021-01040-8 (PMC8317339; doi:10.1186/s12920-021-01040-8)
Supplement: Supplementary file 2 — Additional file 2: Figure S1. Comparison of the error rates between duplicates. The mean error rates across the 12 substitution classes between duplicate experiments are shown as box plots. [file 12920_2021_1040_MOESM2_ESM.docx]

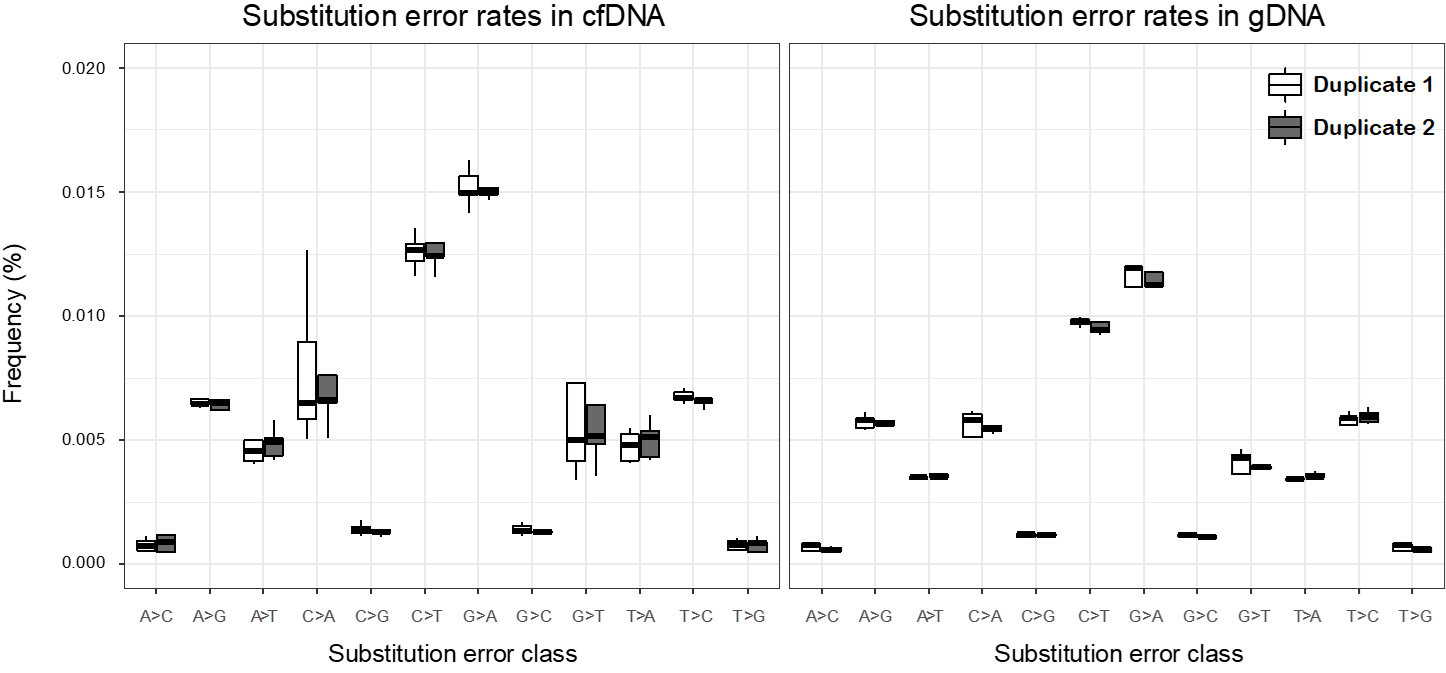


**Supplementary Figure S1. Comparison of the error rates between duplicates.** The mean error rates across the 12 substitution classes between duplicate experiments are shown as box plots.
