# Supplementary material for: Characterization of DNA lesions associated with cell-free DNA by targeted deep sequencing
Source: BMC Med Genomics. 2021 Jul 28;14:192. doi: 10.1186/s12920-021-01040-8 (PMC8317339; doi:10.1186/s12920-021-01040-8)
Supplement: Supplementary file 3 — Additional file 3: Figure S2. Comparison of error rates in the sequencing data of cfDNA and cellular gDNA samples. The box plots display the distribution of mean error rates (left panel) in the 12 substitution classes and (right panel) in total from the cfDNA and cellular gDNA samples. While the data in Figure 1 were obtained from healthy volunteers, the data shown in this figure were obtained from lymphoma patients. [file 12920_2021_1040_MOESM3_ESM.docx]

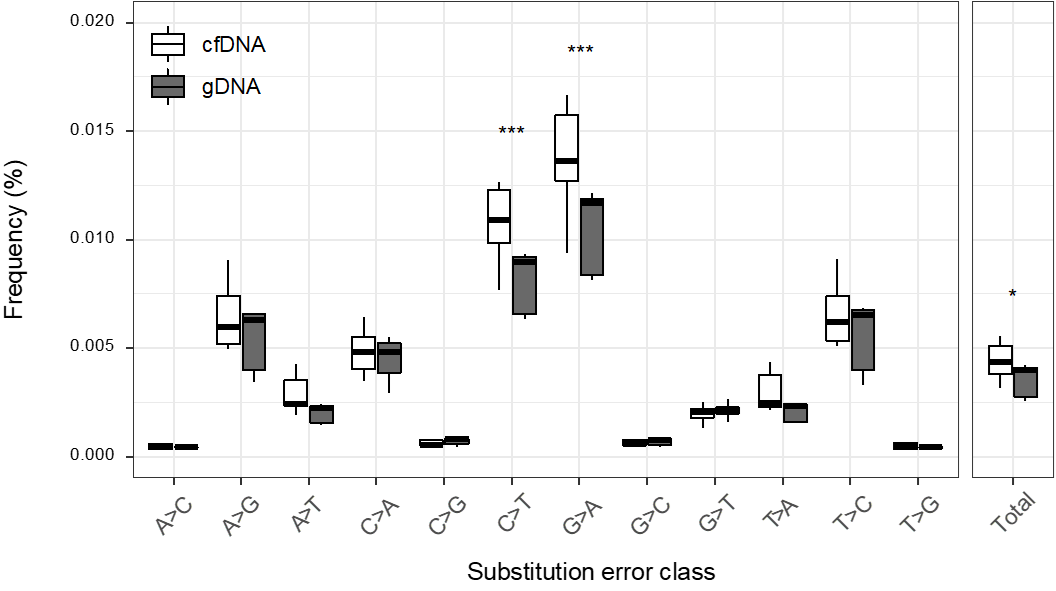


**Supplementary Figure S2. Comparison of error rates in the sequencing data of cfDNA and cellular gDNA samples.** The box plots display the distribution of mean error rates (left panel) in the 12 substitution classes and (right panel) in total from the cfDNA and cellular gDNA samples. While the data in Figure 1 were obtained from healthy volunteers, the data shown in this figure were obtained from lymphoma patients.
