# Supplementary material for: Characterization of DNA lesions associated with cell-free DNA by targeted deep sequencing
Source: BMC Med Genomics. 2021 Jul 28;14:192. doi: 10.1186/s12920-021-01040-8 (PMC8317339; doi:10.1186/s12920-021-01040-8)
Supplement: Supplementary file 4 — Additional file 4: Figure S3. Comparison of 12 substitution error rates between plasma cfDNA and cellular gDNA samples collected in non-fixative EDTA tube. The box plots display the distribution of mean error rates in each substitution error class. ***; Asterisks indicates statistically significant difference (p-value ≤ 0.01). [file 12920_2021_1040_MOESM4_ESM.docx]

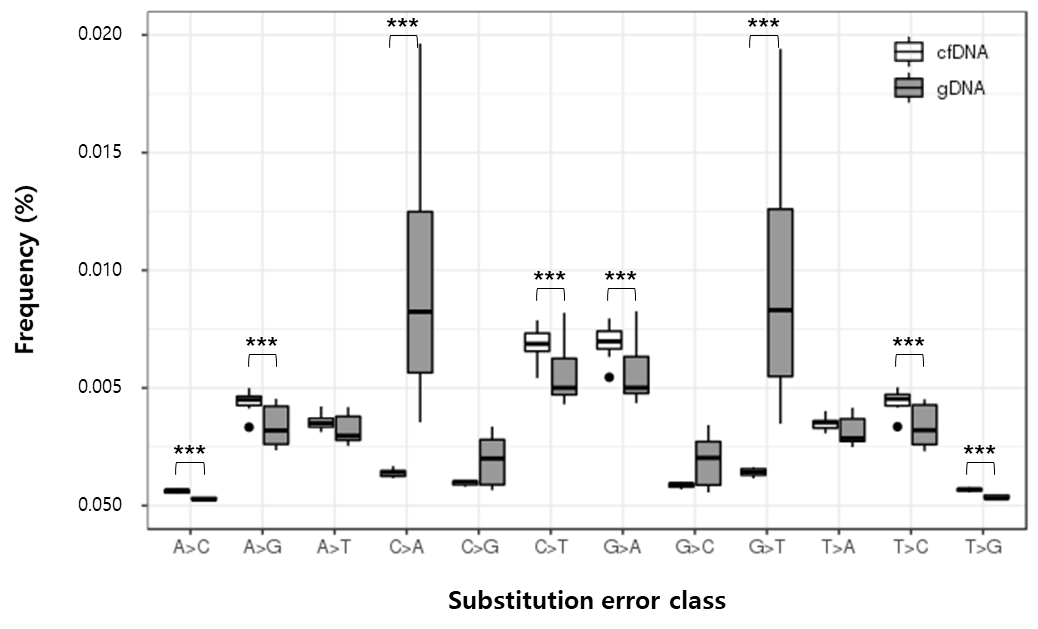


**Supplementary Figure S3. Comparison of 12 substitution error rates between plasma cfDNA and cellular gDNA samples collected in non-fixative EDTA tube.** The box plots display the distribution of mean error rates in each substitution error class. ***; Asterisks indicates statistically significant difference (*p*-value ≤ 0.01)
