# Supplementary material for: Characterization of DNA lesions associated with cell-free DNA by targeted deep sequencing
Source: BMC Med Genomics. 2021 Jul 28;14:192. doi: 10.1186/s12920-021-01040-8 (PMC8317339; doi:10.1186/s12920-021-01040-8)
Supplement: Supplementary file 5 — Additional file 5: Figure S4. Background error rates near the DNA break point. The error rates for the first three 5 bp-bins near the DNA break points from the cfDNA and gDNA samples were calculated and compared. Data generated independently from (a) healthy volunteers and (b) lymphoma patients show similar patterns across all substitution classes. [file 12920_2021_1040_MOESM5_ESM.docx]

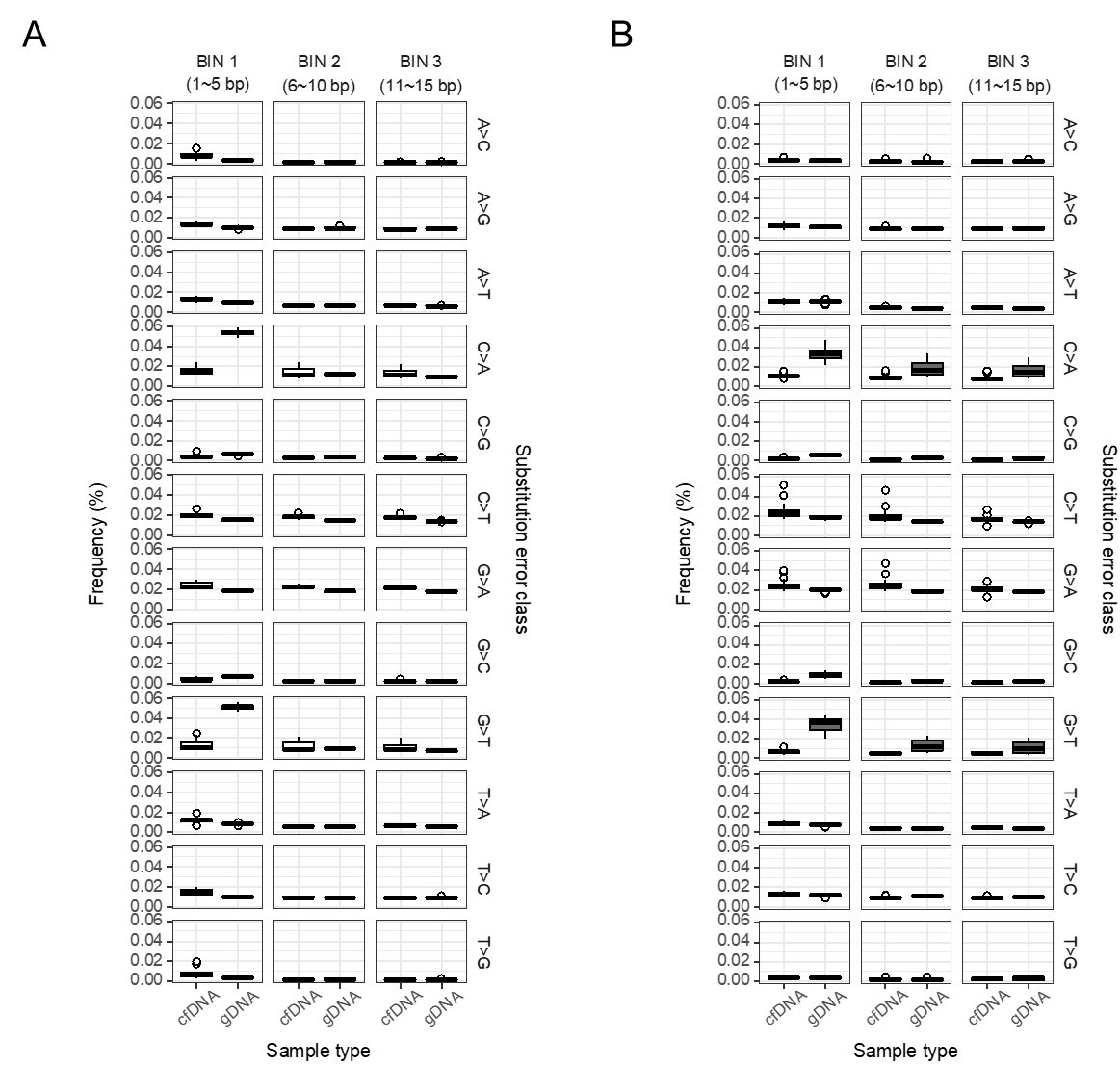


**Supplementary Figure S4. Background error rates near the DNA break point.** The error rates for the first three 5 bp-bins near the DNA break points from the cfDNA and gDNA samples were calculated and compared. Data generated independently from **(a)** healthy volunteers and **(b)** lymphoma patients show similar patterns across all substitution classes.
