# Supplementary material for: Characterization of DNA lesions associated with cell-free DNA by targeted deep sequencing
Source: BMC Med Genomics. 2021 Jul 28;14:192. doi: 10.1186/s12920-021-01040-8 (PMC8317339; doi:10.1186/s12920-021-01040-8)
Supplement: Supplementary file 6 — Additional file 6: Figure S5. Differences in error rates due to DNA fragmentation. The mean error rates across the 12 substitution classes are shown as box plots for the mild shearing condition (left panel) and the standard condition (right panel). [file 12920_2021_1040_MOESM6_ESM.docx]

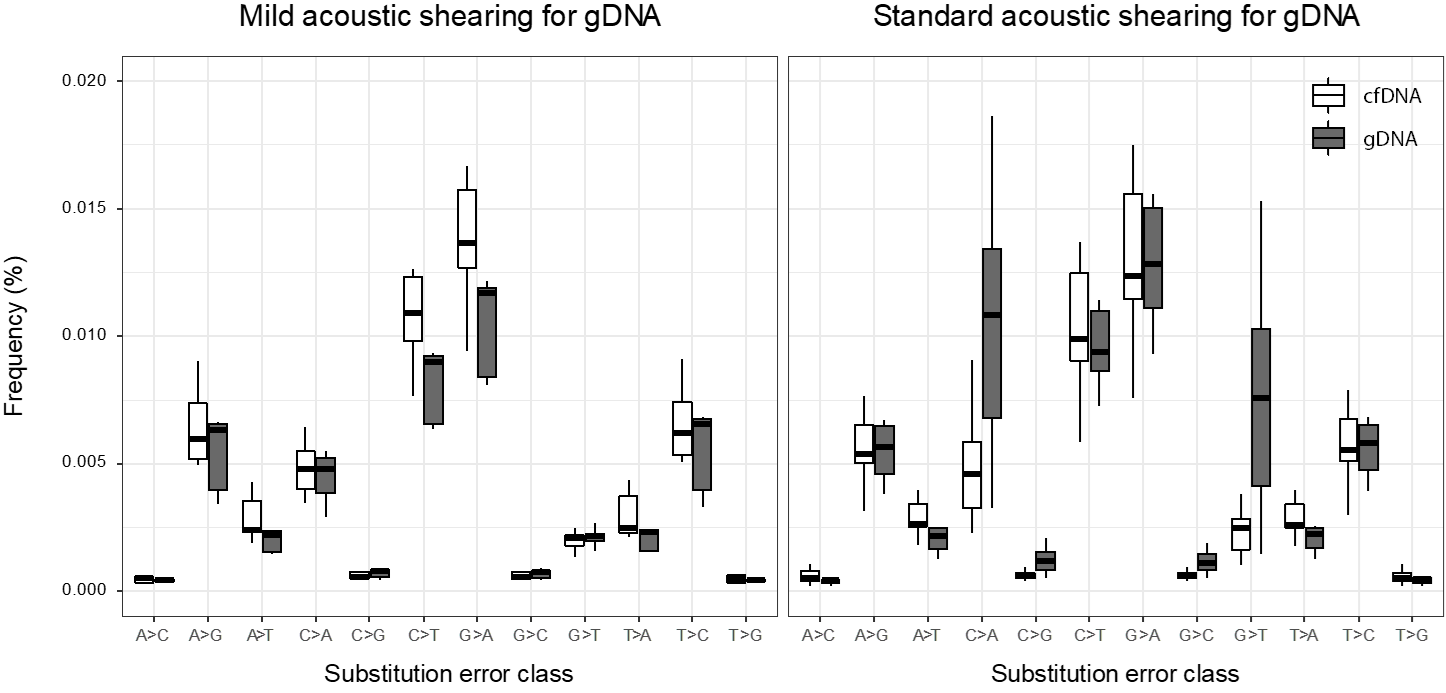


**Supplementary Figure S5. Differences in error rates due to DNA fragmentation.** The mean error rates across the 12 substitution classes are shown as box plots for the mild shearing condition (left panel) and the standard condition (right panel).
